# Conversion of sugar beet residues into lipids by *Lipomyces starkeyi* for biodiesel production

**DOI:** 10.1186/s12934-020-01467-1

**Published:** 2020-11-10

**Authors:** Francesca Martani, Letizia Maestroni, Mattia Torchio, Diletta Ami, Antonino Natalello, Marina Lotti, Danilo Porro, Paola Branduardi

**Affiliations:** Department of Biotechnology and Biosciences, University of Milano Bicocca, 20126 Milan, Italy

**Keywords:** *Lipomyces starkeyi*, Sugar beet pulp, Molasses, Tags, Fames, Biodiesel, Biorefinery

## Abstract

**Background:**

Lipids from oleaginous yeasts emerged as a sustainable alternative to vegetable oils and animal fat to produce biodiesel, the biodegradable and environmentally friendly counterpart of petro-diesel fuel. To develop economically viable microbial processes, the use of residual feedstocks as growth and production substrates is required.

**Results:**

In this work we investigated sugar beet pulp (SBP) and molasses, the main residues of sugar beet processing, as sustainable substrates for the growth and lipid accumulation by the oleaginous yeast *Lipomyces starkeyi*. We observed that in hydrolysed SBP the yeast cultures reached a limited biomass, cellular lipid content, lipid production and yield (2.5 g/L, 19.2%, 0.5 g/L and 0.08 g/g, respectively). To increase the initial sugar availability, cells were grown in SBP blended with molasses. Under batch cultivation, the cellular lipid content was more than doubled (47.2%) in the presence of 6% molasses. Under pulsed-feeding cultivation, final biomass, cellular lipid content, lipid production and lipid yield were further improved, reaching respectively 20.5 g/L, 49.2%, 9.7 g/L and 0.178 g/g. Finally, we observed that SBP can be used instead of ammonium sulphate to fulfil yeasts nitrogen requirement in molasses-based media for microbial oil production.

**Conclusions:**

This study demonstrates for the first time that SBP and molasses can be blended to create a feedstock for the sustainable production of lipids by *L. starkeyi*. The data obtained pave the way to further improve lipid production by designing a fed-batch process in bioreactor.

**Graphical abstract:**

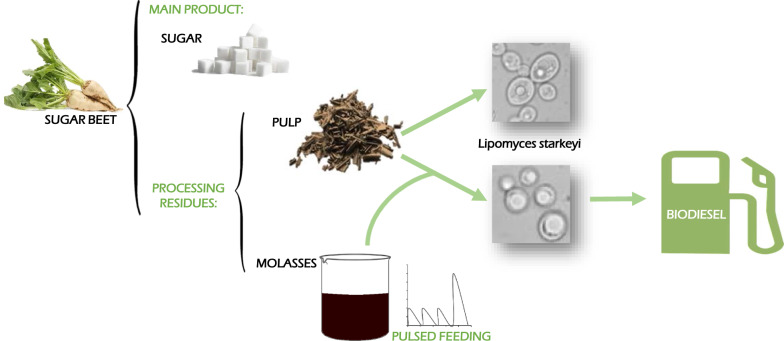

## Background

The depletion of fossil reserves, the environmental concerns about greenhouse gas (GHG) emissions and the increasing demand for energy are boosting the conversion from a fossil-based economy into a bio-based economy. In this context, biodiesel is receiving an ever-increasing attention as an alternative fuel source because of its renewability, biodegradability, non-toxicity and lower emission of pollutants compared to petro-diesel [1]. In addition, biodiesel is safer for transportation, distribution and handling than petroleum diesel, and blends up to 20% of biodiesel can be used in current engines without modifications [1]. This “green” fuel is obtained from oils through a transesterification reaction where triacylglycerols (TAGs) react with a short-chain alcohol (typically methanol) in the presence of a catalyst to form fatty acid alkyl esters (FAAEs).

The main feedstocks used for the production of biodiesel are vegetable oils, animal fats and waste cooking oils. Vegetable oils are considered the most promising due to their characteristics, composition and abundance: however, due to the food versus fuel debate they cannot fulfil the requirement for sustainable biodiesel production in large scale [2]. Microbial lipids, also called single-cell oils (SCOs), are considered as potential alternatives to vegetable oils to produce biodiesel [3]. In fact, compared to vegetable oils, SCOs offer different advantages, such as no competition (direct or indirect) with food production, less labour requirement, short life cycle, and independence of season and climate [4, 5]. Lipid biosynthesis in oleaginous microorganisms occurs under specific nutrient-deficient conditions [5]. One of the common strategies used to boost lipid accumulation is the cultivation in media with high C/N ratio: when nitrogen is limiting the excess of carbon present in the medium is directed into the biosynthesis of lipids [6].

Oleaginous yeasts are prominent platforms to produce SCOs because of their fast growth and high accumulation of lipids (up to the 70% of dry weight) with a composition similar to vegetable oils [7]. Among them, the oleaginous yeast *Lipomyces starkeyi* is considered an emerging cell factory for the production of SCOs due to its ability to accumulate high amounts of lipids and to utilize a variety of carbon and nitrogen sources [8].

One of the main factors limiting the use of SCOs as a concrete alternative to vegetable oils is the high cost associated with the substrates used for media formulation. The use of glucose as feedstock has been estimated to represent about the 40% of the total costs of production of yeast SCOs [9]. Therefore, the development of viable and economically competitive processes to produce SCOs requires the exploitation of raw and residual organic materials that are available in large quantities and at low cost.

Sugar beet refinery produces a huge amount of residues in the form of molasses and sugar beet pulp (SBP), whose worldwide production in 2018/2019 has been estimated to be about 65 and 4 million tonnes, respectively [10]. Molasses can be used as animal feed and as a feedstock for bioethanol production; its high carbon (mostly sucrose) and poor nitrogen content makes molasses also a promising substrate for microbial lipids, but its utilization requires the addition of external nitrogen sources [11–14]. SBP can be used as animal feed but it is considered a potential feedstock for microbial productions because the high fraction of carbohydrates (24–32% hemicellulose, 22–30% cellulose and 38–62% pectin) [15, 16] can promote microbial growth and the low lignin content (< 2%) can reduce the costs of pre-treatment, compared to most other lignocellulosic biomasses. The use of SBP as substrate for the production of bioethanol has been described in different papers [16–18]. However, the utilization of SBP for microbial oil production may be limited by its low (20–40) C/N ratio [19–21]. Indeed, lipid accumulation can be achieved by using media with a high C/N ratio [6] and from literature we know that the optimum C/N ratio for lipid accumulation within *L. starkeyi* cells is in the range of 50–150 [22–24], depending on media and growth conditions. Wang and colleagues reported the potential of SBP for lipid production by the oleaginous yeasts *Trichosporon cutaneum, T. fermentas and Cryptococcus curvatus* by using a fermentation medium in which SBP hydrolysate was added with yeast extract and some salts [25]. Despite its unfavourable composition for microbial lipid production, if combined with molasses SBP has the potential to fulfil the deficiency of nitrogen just enough to trigger microbial oil production, and to provide additional carbon sources.

In this work we investigated the possibility to use sugar beet molasses and SBP deriving from sugar beet processing as sole carbon and nitrogen sources to support lipogenesis in the oleaginous yeast *L. starkeyi*. We could also demonstrate that SBP hydrolysate better supported growth and lipid production on molasses compared with ammonium sulphate, which is commonly used to fulfil yeasts nitrogen requirement. In addition, the application of a molasses pulse-fed cultivation allowed a better lipid production compared to the batch culture.

## Results

### Growth and lipid accumulation by *L. starkeyi* in SBP hydrolysates

**Fig. 1:**
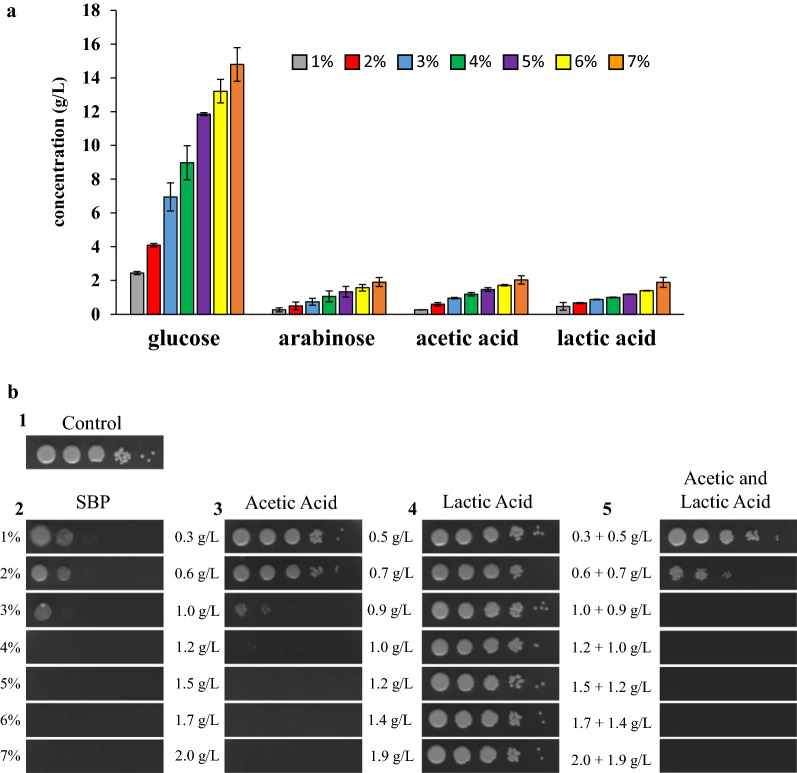
*L. starkeyi* and SBP hydrolysates. **a** Concentration of glucose, arabinose, acetic acid and lactic acid in SBP hydrolysates generated at increasing percentages of TS after 72 h of enzymatic hydrolysis. Data shown as mean ± standard deviation. Error bars correspond to standard deviation of triplicate samples. **b** Spot assay of *L. starkeyi* precultured for 24 h on plates containing minimal media (**b1**), different concentration of SBP hydrolysate (**b2**) and on plates with minimal media containing different concentration of acetic acid (**b3**), lactic acid (**b4**) and both acetic and lactic acid (**b5**). The first drop corresponds to 5 µL of cells at OD_660nm_1, followed by serial 1/10 dilutions. Plates were incubated at 30 °C and observed after 3 days, when pictures were taken. Data shown are representative of three independent experiments

When lignocellulosic hydrolysates are used as substrate for microbial productions, it is necessary to find a compromise between the need to start from a high sugar concentration and the necessity to minimize the toxic effect of inhibitors. Indeed, during pre-treatments not only sugars are released but also inhibitors like weak organic acids. In our case, we noticed the presence of acetic and lactic acid (Fig. 1a), which can be mainly ascribable to the initial contamination of the SBP with acid-producing bacteria, as reported by Kühnel and colleagues [26].

To test the toxic effects of SBP hydrolysate and of acetic and lactic acid on the growth of *L. starkeyi* we carried out different spot assays. Cells were spotted on solid SBP media and on solid minimal media added with acetic or lactic acid in the corresponding concentrations as present in SBP hydrolysates. SBP hydrolysates originated from 1 to 7% of initial total solid (TS) (Fig. 1b). The higher was the concentration of SBP hydrolysate, the higher was the inhibition of *L. starkeyi*’s growth, which was possible until 3% SBP (Fig. 1b.2). Acetic acid showed to cause a strong inhibition on cell growth starting from the concentration of 1 g/L (Fig. 1b.3). On the contrary, no inhibition was observed for lactic acid at all the tested concentrations (Fig. 1b.4). When both weak organic acids were combined (Fig. 1b.5), the growth inhibition was similar to the one observed in the presence of the corresponding SBP hydrolysate.

**Fig. 2:**
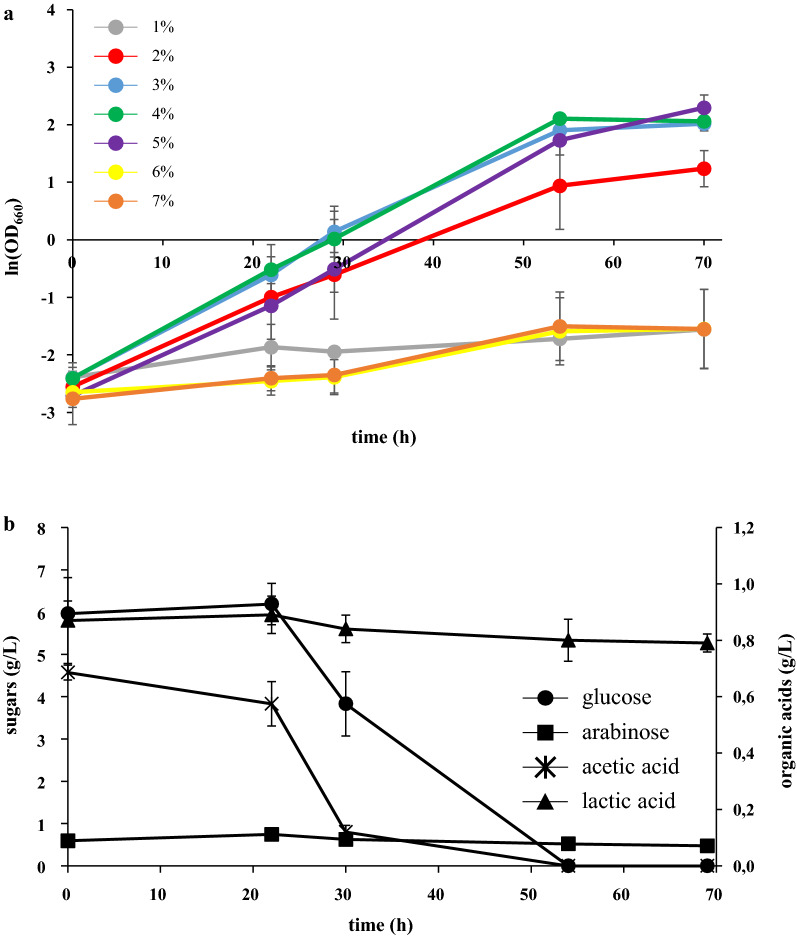
*L. starkeyi* growth in SBP hydrolysates. **a** Growth curves of *L. starkeyi* in SBP hydrolysates generated at increasing percentages of TS. **b** Glucose, arabinose, acetic acid and lactic acid concentration (g/L) in the culture medium during the first 72 h of cultivation in 3% SBP hydrolysate. Data shown as mean ± standard deviation. Error bars correspond to standard deviation of triplicate samples

With the aim to better determine the proper concentration of SBP to be used, *L. starkeyi* was grown in SBP hydrolysates from 1 to 7% TS. Poor or no growth was observed at low (1 and 2% TS) and high (6 and 7% TS) concentrations of SBP hydrolysates (Fig. 2a and Additional file 1: Table S1). At low TS percentages, sugars content is probably not enough to sustain growth, whereas at high TS percentages acetic acid is reaching an inhibitory concentration (Fig. 1). 3, 4 and 5% TS supported yeast growth without apparent impairment due to inhibitors, allowing the cultures to reach a comparable final OD and µ after 72 h of growth (Fig. 2a and Additional file 1: Table S1). Because of the heterogeneous nature of this raw substrate, the use of diverse SBP stocks can generate hydrolysates with different amounts of sugars and inhibitors, despite the precaution taken for standardising as much as possible the procedure. To avoid possible growth delay or inhibition due to this heterogeneity, and consequent discrepancies, we selected 3% SBP hydrolysate as a suitable medium for reproducible *L. starkeyi* cultivation. Moreover, on solid media 3% SBP hydrolysate is the higher concentration where we observed growth (Fig. 1b.2).

The capability of *L. starkeyi* to metabolize glucose, arabinose, lactic acid and acetic acid present in SBP hydrolysates was determined by analysing the concentration of these compounds at different time points. As previously observed [24, 27, 28], *L. starkeyi* is able to co-consume glucose and acetic acid, but arabinose and lactic acid were not assimilated throughout the cultivation time (Fig. 2b).

Under these conditions, cells accumulated about 19.2% of their dry weight as intracellular oils, leading to a production of 0.5 g/L of lipids after 144 h (Table 1). These values are low if compared with the results obtained by cultivating the same strain on other residual substrates [29–31]: very likely this depends on the low C/N ratio of the media, which is not suitable to efficiently redirect the yeast metabolism towards lipid biogenesis.

**Table 1:** Biomass and lipid production by *L*. *starkeyi*

Cultivation mode	Molasses (%)	SBP (%)	(NH_4_)_2_SO_4_ (g/L)	CDW (g/L)	Lipid production (g/L)	Lipid content (%)	Lipid yield (g_l_/g_s_)
Batch	-	3	–	2.5 ± 0.4	0.5 ± 0.3	19.2 ± 4.2	0.080 ± 0.039
	6	3	–	19.7 ± 1.2	9.3 ± 0.3	47.2 ± 2.6	0.167 ± 0.006
	6	–	0.5	5.0 ± 1.3	2.0 ± 1.0	30.2 ± 3.8	ND
	6	–	1.0	4.2 ± 0.4	1.9 ± 0.7	36.4 ± 5.1	ND
	6	–	2.0	2.2 ± 0.6	0.9 ± 0.1	29.6 ± 2.7	ND
Pulsed fed-batch	6	3	–	20.5 ± 2.2	9.7 ± 0.7	49.2 ± 0.9	0.178 ± 0.012
	6	–	0.5	15.6 ± 0.1	6.1 ± 0.3	38.3 ± 2.6	ND
	6	–	1.0	16.2 ± 0.1	6.7 ± 0.4	38.9 ± 3.8	ND
	6	–	2.0	16.1 ± 0.5	6.9 ± 0.6	41.0 ± 4.4	ND

Cells were grown in 6% molasses with the addition of 3% SBP hydrolysate or (NH_4_)_2_SO_4_ in batch and pulsed fed-batch cultures. CDW, lipid production, lipid content and lipid yield were calculated after 144 h of growth. Lipid yield (g_l_/g_s_) was calculated considering the amount of glucose, sucrose and acetic acid consumed by cells

*ND* not determined

### Growth and lipid accumulation in SBP hydrolysate blended with molasses

**Fig. 3:**
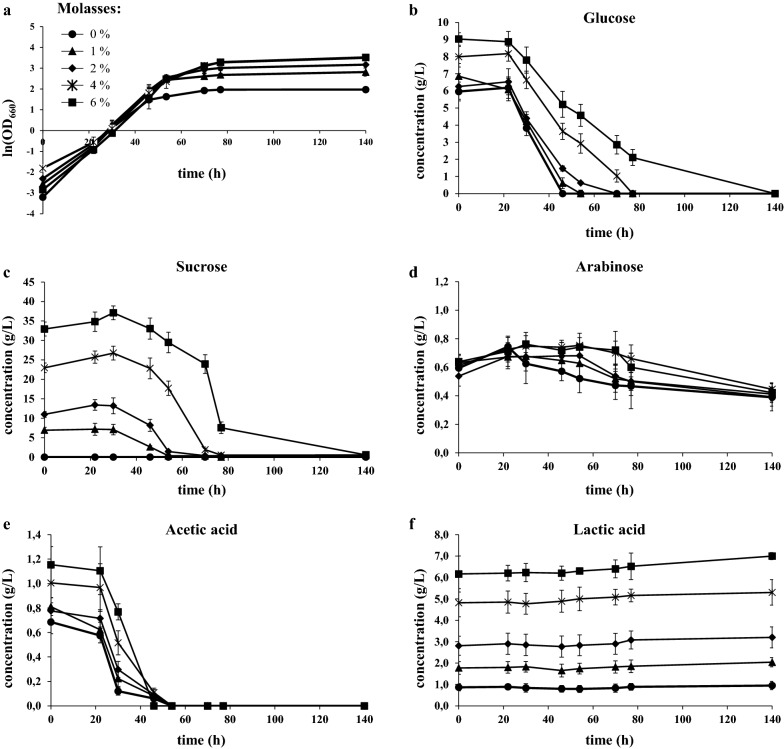
*L. starkeyi* growth in molasses blended with 3% SBP hydrolysate. **a** Growth curves of *L. starkeyi* in SBP hydrolysates generated at 3% TS and 0%, 1%, 2%, 4% or 6% of molasses. Glucose (**b**), sucrose (**c**), arabinose (**d**), acetic acid (**e**) and lactic acid (**f**) concentrations during growth of *L. starkeyi* in SBP hydrolysate generated at 3% TS and 0%, 1%, 2%, 4% or 6% of molasses. Data shown as mean ± standard deviation. Error bars correspond to standard deviation of triplicate samples

To unbalance the C/N ratio, we evaluated the effect of the addition of different concentrations of molasses to 3% SBP hydrolysate on *L. starkeyi* growth and lipid accumulation. As shown in Fig. 3a, the final OD reached by cultures directly correlated with the concentration of molasses up to 4% molasses. At 4% and 6% molasses cells grow to a comparable final OD and this situation could be caused by the presence in 6% molasses of moderately-toxic amount of acetic acid (Fig. 3e, square symbol) and/or by the high sucrose concentration that might cause osmotic stress (Fig. 3c, square symbol). In respect to main metabolites, it can be observed that cells co-consumed glucose and acetic acid from about 24 h of cultivation and started to also co-consume sucrose after about 48 h (Fig. 3b, c, e), as also reported in literature [24]. As observed in SBP hydrolysate alone, arabinose and lactic acid were not metabolised (Fig. 3d, f).

**Fig. 4:**
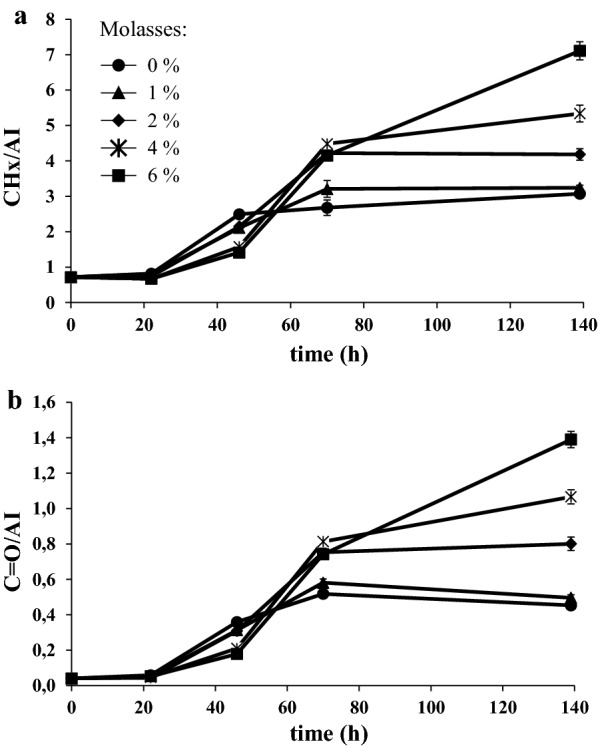
Fatty acid production in molasses blended with 3% SBP hydrolysate. CH stretching band area (**a**) and ester C=O (**b**) measured over time by Fourier transform infrared (FTIR) of *L. starkeyi* cells growing in SBP hydrolysates generated at 3% TS and 0%, 1%, 2%, 4% or 6% of molasses. Values were normalized for the total protein content given by the amide I band (AI) area. Data shown as mean ± standard deviation. Error bars correspond to standard deviation of triplicate samples

Lipid accumulation in intact cells of *L. starkeyi* grown in SBP hydrolysate added with molasses was monitored over time by Fourier Transform Infrared (FTIR) micropectroscopy. FTIR is an efficient tool for rapidly following up microbial lipid accumulation at different stages of growth [32–39], since it can analyze intact cells identifying specific molecular groups by their absorption bands. Starting from the spectra obtained sampling the cultivations over time, Fig. 4 illustrates the temporal evolution of the CHx stretching band area, between 3050 and 2800 cm^−1^, and of the ester carbonyl band area, between 1760 and 1730 cm^−1^, after normalization for the total protein content, given by the amide I band area (Additional file 1: Figure S1). The analysis showed that the addition of molasses at concentrations higher than 1% significantly improved the final intracellular lipid production (Fig. 4). In the presence of 6% molasses, intracellular lipids were the 47.2% of cellular dry weight and lipid production reached 9.3 g/L after 144 h (Table 1).

**Fig. 5:**
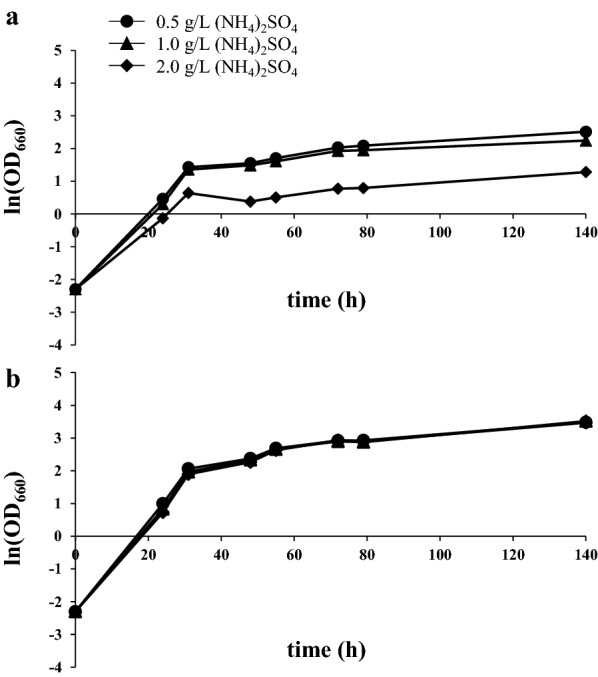
*L. starkeyi* growth in molasses blended with (NH_4_)_2_SO_4_. Growth curves of *L. starkeyi* in molasses and 0.5, 1.0 or 2.0 g/L of (NH_4_)_2_SO_4_ in batch **(a)** and pulsed-fed batch cultures **(b)**. Data shown as mean ± standard deviation. Error bars correspond to standard deviation of triplicate samples

From these data it seems that SBP can be considered more as a nitrogen supplementation than as a carbon source. Indeed, we determined nitrogen content in 3% SBP, which is 27.0 mg/L, 17.5 mg/L from primary amino acids and 9.5 mg/L from ammonia. Ammonium sulphate is one of the compounds commonly used to fulfil the need of nitrogen in yeast media formulations [40, 41]. We therefore formulated a medium where ammonium sulphate at different concentrations was blended with molasses to compare *L. starkeyi* growth and lipid accumulation in the presence of SBP or ammonium sulphate as nitrogen source. As in sugar beet pulp the nitrogen is present mainly in its organic form, we supplemented the medium with a higher ammonium sulphate concentration [13, 14], since it represents a less bioavailable inorganic nitrogen source. Despite the higher availability of nitrogen, we observed that growth was significantly reduced in the presence of all the (NH_4_)_2_SO_4_ concentrations (Fig. 5a) if compared with the growth observed using only residual biomasses (Fig. 3a). As a consequence, we observed lower values of lipid production, even after 144 h of cultivation (Table 1). SBP hydrolysate therefore resulted to be superior to ammonium sulphate in supporting growth and lipogenesis in *L. starkeyi* when blended with molasses.

### Growth and lipid accumulation in molasses pulse-fed batch cultures

FTIR data on lipid accumulation in SBP hydrolysate added with molasses showed that lipid accumulation was faster at low concentrations of molasses (1% and 2%), or even in its absence, during about the first 48 h of growth (Fig. 4). Because of the existence of a correlation between lipid accumulation and cell density that was independent from the concentration of molasses (Additional file 1: Figure S2), the slightly delayed accumulation of lipid observed in the presence of 6% molasses might be a consequence of the slowed growth shown in Fig. 3a and discussed above.

**Fig. 6:**
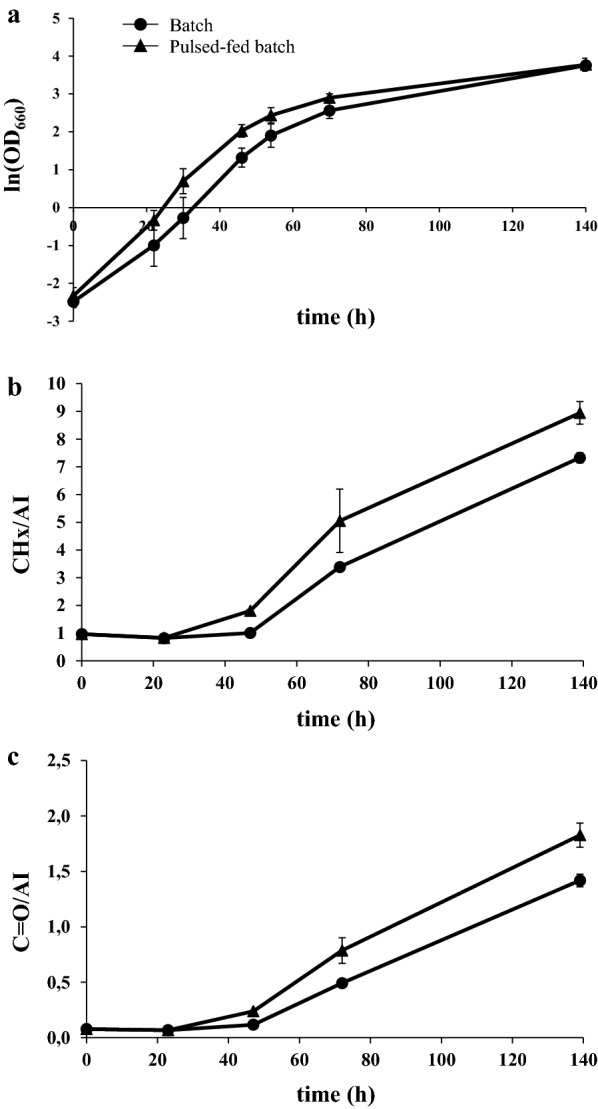
Comparison of growth and fatty acid production by *L. starkeyi* growing in molasses blended with SBP hydrolysate in batch and pulsed-fed batch cultures. **a** Growth curves of *L. starkeyi* in 6% molasses and 3% SBP hydrolysate in batch and pulsed-fed batch cultures. CH stretching band area (**b**) and ester C=O (**c**) measured over time by Fourier transform infrared (FTIR) of *L. starkeyi* cells growing in 6% molasses and 3% SBP hydrolysate in batch and pulsed-fed batch cultures. Values were normalized for the total protein content given by the amide I band (AI). Data shown as mean ± standard deviation. Error bars correspond to standard deviation of triplicate samples

The use of pulsed feeding fermentations has the advantage to avoid the possible stress imposed by high concentrations of substrates and to temporally separate cell growth and lipid accumulation, which has been described as a successful method to obtain high lipid production [42, 43]. Considering this, we cultivated cells in SBP hydrolysate with pulse feeding of molasses at different time intervals (0, 24 h and 48 h) and low concentrations (1%), followed by a pulse feeding at 72 h with a higher molasses concentration (3%). When a pulse feeding of molasses was applied, we observed a faster growth during the first hours compared with the batch culture (Fig. 6a and Additional file 1: Table S1). At the end of the fermentation, cells reached a similar OD (Fig. 6a; Table 1) and consumed almost all sucrose, glucose and acetic acid (Additional file 1: Figure S3).

FTIR measurements showed that the pulse-feeding cultivation allowed a higher intracellular lipid accumulation compared to the batch culture throughout all the fermentation (Fig. 6b and c). The ratio between the lipid accumulation measured by FTIR and cell density, as a matter of fact, was higher in fed-batch compared with batch cultures (Additional file 1: Figure S2), confirming that molasses pulse feeding is effective in increasing the intracellular lipid content.

The difference in lipid accumulation among batch and fed-batch cultures was not evident when lipid content was measured as the ratio of their weight to dried cell biomass (lipid content %, Table 1), but we cannot exclude that lipid extraction was not completely efficient. In fact, compared to lipid extraction technologies, spectroscopic analysis have the advantage to evaluate lipid accumulation in intact cells avoiding possible lipid loss [32]. Moreover, the addition of molasses increased not only lipid production but also lipid yield (g/g), and this improvement was more pronounced in pulse-fed compared to batch cultivations (Table 1).

Molasses fed-batch fermentations were also applied using ammonium sulphate as an external nitrogen source. Under these conditions, the growth of *L. starkeyi* was better than in batch cultures and was not affected by the concentration of ammonium sulphate (Fig. 5b, Table 1). After 144 h of growth, cell dry weight, lipid production and lipid content were not affected by the different concentrations of ammonium sulphate. These performances were higher than in batch cultures, but lower than in molasses pulsed-fed cultivations with SBP hydrolysate (Table 1).

### Composition of biodiesel deriving from oils produced by *L. starkeyi* on sugar beet residues

The lipids produced by *L. starkeyi* in SBP hydrolysate without and with molasses in batch and fed-bath cultures were transmethylated by alkaline catalysis and the resulting fatty acid methyl esters (FAMEs) were determined by gas chromatography. The main fatty acids produced by *L. starkeyi* in SBP hydrolysate with or without molasses were oleic (18:1) and palmitic (16:0) acids (Table 2). If compared with the composition of fatty acids of cells grown in SBP hydrolysate alone, the addition of molasses increased the percentage of palmitic acid (16:0) and reduced the percentage of linoleic acid (18:3), overall increasing and reducing the content of monounsaturated and polyunsaturated fatty acids, respectively (Table 2).

If on one hand microbial lipids are emerging as important platforms for the sustainable production of biodiesel, on the other hand immobilized lipases are considered as alternative catalyst of transesterification reactions that are consistent with the development of green processes [44–46]. For this reason, we evaluated the FAMEs profile deriving from lipids produced by *L. starkeyi* in SBP hydrolysate fed with molasses also using the immobilized lipase Novozym 435. Due to the inhibitory effect of methanol on lipase activity [46, 47], a small amount of methanol (2.5%) was added to the reaction every 24 h for four times (Additional file 1: Figure S4). Compared to the alkaline catalysis, the enzymatic reaction was less efficient and more time consuming (144 h to reach about 65% of FAMEs; Additional file 1: Figure S4). Considering the transesterification, the use of lipase reduced the percentage of linoleic acid (18:2) and increased that of stearic acid (18:0) (Table 2).

**Table 2:** Fatty acid profile of *L. starkeyi*

Cultivation mode	Molasses (%)	SBP (%)	(NH_4_)_2_SO_4_	Catalyst	C14:0	C16:0	C16:1	C18:0	C18:1	C18:2	C18:3	M	P	S	CN*
Batch	–	3	–	NaOH	0.34 ± 0.08	31.7 ± 3.7	6.5 ± 0.2	2.4 ± 0.3	43.8 ± 4.4	8 ± 3.4	0.43 ± 0.1	53.4 ± 0.1	8.4 ±3.6	38.2 ± 3.5	61.2
	6	3	–	NaOH	0.32 ± 0.04	33.2 ± 1	4.9 ± 0.6	3.5 ± 0.6	51.9 ± 0.5	2.8 ± 0.6	< 0.1	57.7 ± 0.3	3 ± 0.4	39.3 ± 0.1	62.9
Pulsed fed-batch	6	3	–	NaOH	0.38 ± 0.05	32.7 ± 1.9	4.4 ± 0.5	3.8 ± 0.5	52.5 ± 3	3.3 ± 0.9	< 0.1	57.9 ± 3.4	3.3 ± 0.8	38.7 ± 2.5	62.9
	6	3	–	Novozym 435	0.45 ± 0	34.2 ± 0.5	4.8 ± 0.2	5 ± 0.3	51.6 ± 1	1.5 ± 0.1	< 0.1	56.4 ± 1.2	1.8 ± 0.2	41.9 ± 0.9	63.6
	6	–	0.5 g/L	NaOH	0.26 ± 0.03	33.9 ± 0.5	2.8 ± 0.1	5.2 ± 0.3	50 ± 1.1	6.3 ± 0.6	< 0.1	53.4 ± 1.1	6.5 ± 0.6	40.1 ± 0.8	62.7

Fatty acids produced by *L. starkeyi* on 3% SBP hydrolysate with or without the addition of 6% molasses in batch and pulsed fed-batch cultures

*M* monounsaturated fatty acids, *P* polyunsaturated fatty acids, *S* saturated fatty acids

*CN was calculated using the equation: CN = 61.1 + 0.088(% 14:0) + 0.133(% 16:0) + 0.152(% 18:0) – 0.101(% 16:1) – 0.039(% 18:1) – 0.243(% 18:2) – 0.395(% 18:3) [48]

## Discussion

In this work, we showed that SBP can be blended with molasses to create a sustainable feedstock that can be efficiently converted into microbial lipids by the oleaginous yeast *L. starkeyi*. To the best of our knowledge, the use of the sole two main streams of residues of the sugar beet processing for sustaining microbial oil production was never reported before. SBP hydrolysate can therefore represent a renewable and low-cost substrate to fulfil the nitrogen requirement of yeasts converting a carbon-rich feedstock, such as molasses or crude glycerol, into SCOs. In such a way, one of the goals of circular economy, which is the depletion of residues and waste, can be addressed.

Different studies demonstrated that the application of two stage cultures is advantageous compared with single-stage cultures for microbial lipid accumulation, thanks to the spatial and temporal separation of a first phase in which cells reach high cell density in a nitrogen enriched medium, and a second phase where cells accumulate lipids in a nitrogen limited medium with excess of carbon [43, 49, 50]. In this work, the availability of molasses achieved by pulsed-fed fermentations in shake flasks resembled the principles of two stage cultures and, accordingly, enabled a higher intracellular lipid accumulation compared with batch fermentations, leading to an average of 9.7 g/L and 0.178 g/g of lipid production and yield, respectively (Table 1).

Our experiments show that addition of molasses also influenced the lipid profile of *L. starkeyi* by reducing the content of polyunsaturated fatty acids and by increasing that of monounsaturated ones (Table 2). This result confirmed that the carbon source provided to oleaginous yeasts can affect the level of saturation of the fatty acids produced, as observed in other works [51, 52], and this in turn can affect several properties of biodiesel. According to the equation reported in [48], the biodiesel produced by the lipids accumulated when *L. starkeyi* is cultivated in SBP hydrolysate blended with molasses had a higher cetane index compared to that obtained from the SBP hydrolysate alone (Table 2). The cetane number is an indicator of the ignition properties of a fuel: the higher the cetane number is, the shorter the ignition delay time is, lowering the emission of nitrogen oxides (NOx) [53]. The cetane number was even higher when the immobilized lipase Novozym 435 was used for the transesterification of microbial lipids (Table 2). This can be ascribable to the slightly reduced percentage of polyunsaturated FAMEs produced by the enzymatic reaction compared to the chemical one; since lipases can show different substrate specificity [54], this difference is very likely ascribable to the fatty acid selectivity of the used lipase. The selection of the catalyst has therefore an important role in determining the final FAMEs composition, but it is important to consider that lipase-mediated reactions still present limitations among which the efficiency of the catalytic reaction and the overall costs [55, 56].

## Conclusion

In this work we described for the first time that the two main residues of sugar beet processing can be used together for lipid production by the oleaginous yeast *L. starkeyi*. We also performed a preliminary assessment to determine the negative effects caused on growth by the principal weak organic acids present in the residues. In the light of the results obtained by the pulse-fed fermentations in shake flasks and by the spot test revealing acetic acid as the main inhibitor, we are currently designing the scale-up of the process in bioreactor. A continuous feeding strategy will be applied, and productivity determined, making possible a preliminary economic evaluation. In conclusion, the valorisation of uneven residual biomasses expands the combination of bioprospecting, strain engineering, and media composition for modulating lipid profile, therefore transforming a problem into possible solutions, considering the applicability of microbial oils into many and diverse industrial sectors.

## Methods

### Raw materials

SBP and sugar beet molasses (SBM) were provided by Cooperativa Produttori Bieticoli (CoProB), Minerbio (BO, Italy). The total solid content in the fresh SBP was 26%. SBP was stored at − 20 °C and thawed prior to use. SBP was mixed with distilled water at the concentration of 250 g/L (65.5 g/L dry weight) and pre-treated in a lab-scale autoclave at 121 °C for 20 min at a pressure of 1.2 bar. After sterilization, the excess of water was removed.

SBM contained about 800 g/L of sucrose and appeared as a dense, dark brown liquid. It was stored at 4 °C and diluted 1:3 (vv^−1^) with distilled water before the sterilization in autoclave.

### Enzymatic hydrolysis of SBP

Enzymatic hydrolysis was performed using the experimental enzyme mixture NS22-201 (50 µl/g dry weight) kindly provided by Novozymes. Autoclaved SBP was mixed with sodium citrate 100 mM pH 5.5 at TS loading ranging from 10 to 70 g/L dry weight. The reactions were carried out in Erlenmeyer flasks in orbital shaker at 50 °C and 160 rpm for 72 h. The samples were centrifuged at 10,000 rpm for 10 min and the supernatant was recovered and stored at 4 °C.

### Yeast strain, media and growth conditions

*Lipomyces starkeyi* (DSM70295) was purchased from DSMZ. Yeasts were stored in cryotubes at − 80 °C in 20% glycerol (vv^−1^) and pre-grown on YPD agar plates (20 g/l glucose, 20 g/l peptone, 10 g/l yeast extract and 20 g/l agar) for 3 days at 30 °C. Precultures were grown in YPD medium and cultivated for 1 day at 30 °C on an orbital shaker at 220 rpm. Yeast extract was provided by Biolife Italiana S.r.l., Milan, Italy. All the other reagents were provided by Sigma-Aldrich Co., St Louis, MO, USA.

Growth curves were obtained by inoculating yeast cells in SBP hydrolysate at an initial optical density of 0.1 (660 nm) and then the optical density was measured at specific time intervals over at least 72 h from the inoculum. Where specified, molasses was added at the indicated percentage considering 100% the concentration of the non-diluted molasses. Yeasts were grown in shake flasks at 30 °C and 220 rpm and the ratio of flask volume:medium was 5:1.

To determine the Cellular Dry Weight (CDW), the biomass was harvested by centrifugation of the culture samples at 14,000 rpm for 10 min. The pellets were then washed twice with distilled water and dried at 40 °C (Concentrator 52301, Eppendorf, Germany) until a constant weight was obtained.

For each experiment, at least three biological replicates have been performed.

### Spot assay

Spot assay was performed using cells precultured for 24 h in YPD media. From these cultures, 4 series of 1:10 dilution were prepared, starting with an initial optical density of 1 (660 nm). We spotted 5 μl of each of the five suspensions gradually diluted on the different plates and let the cells grow for 3 days at 30 °C. We performed each spot assay in duplicate.

We prepared two different kind of solid media to perform spot assay: one with different concentrations of SBP hydrolysate and one with minimal medium, added with different concentrations of acetic acid and/or lactic acid. As a control, minima medium plates without inhibitors were prepared.

Solid media containing SBP were prepared by mixing agar 2X (40 g/L, autoclaved) with 10% SBP diluted as needed.

The minimal media we used is MeOL medium: 1 g/L of yeast extract, 1.31 g/L of (NH_4_)_2_SO_4_, 0.95 g/L of Na_2_HPO_4_, 2.7 g/L of KH_2_PO_4_, 0.2 g/L of Mg_2_SO_4_·7H_2_O and 20 g/L agar. After the pH was adjusted to 5.5 using NaOH 4 M and autoclaved. The medium was supplemented with a 100X trace mineral stock solution consisting of: 4 g/L CaCl_2_·2H_2_O; 0.55 g/L FeSO_4_·7H_2_O; 0.52 g/L citric acid; 0.10 g/L ZnSO_4_·7H_2_O; 0.076 g/L MnSO_4_·H_2_O; and 100μL 18 M H_2_SO_4_. Glucose and Xylose were added as additional carbon source with a final concentration of 10 g/L each. Acetic and lactic acid were added in MeOL media at the desired concentrations.

### Sugars and acids determination

Samples collected at different time points were centrifuged at 14,000 rpm for 10 min. The supernatants were diluted (when appropriate) in milliQ water and the concentrations of the main metabolites were determined with HPLC using a Rezex ROA – Organic Acid H + (8%) (Phenomenex). The eluent was 0.01 M H_2_SO_4_ pumped at 0.5 mL min^−1^ and column temperature was 40 °C. Separated components were detected by a refractive-index detector and peaks were identified by comparing them with known standards (Sigma Aldrich, St Louis, MO, USA).

### Nitrogen content

Nitrogen content in SBP and SBM is been quantified in terms of primary amino nitrogen from free amino acids and free ammonium ions. We used two kits purchased from Megazyme: Primary Amino Nitrogen Assay Kit (K-PANOPA) and Urea/Ammonia Assay Kit (Rapid) (K-URAMR). We followed instructions in the booklet. At the end of the two assays, we summed together the two contributes in order to have total nitrogen content.

### Lipid extraction and transesterification

Lipid extraction was performed using a method modified from [57]. Briefly, about 1 g of frozen cells was resuspended in 20 ml of HCl at a final concentration of 1 M and incubated for 1 h at 95 °C. The cooled mixture was transferred in a tube and 25 ml of chloroform:methanol (2:1 v/v) were added. After centrifugation at 10,000 rpm for 10 min, the bottom layer (organic fraction) was transferred to a second tube. For the second extraction step, 15 ml of chloroform were added, the mixture was centrifuged at 10,000 rpm for 10 min and the chloroform layer was transferred to the second tube. The amount of total lipids was determined by gravimetric measurement after chloroform was evaporated. The dried samples were stored at 4 °C.

FAMEs were prepared by alkaline catalysis as follows: 5 ml of KOH–methanol 0.4 M were added to 100 mg of microbial lipids and incubated at 60 °C for 1 h. Then, 5 ml of BF_3_-methanol (14% w/w) were added and the mixture was incubated at 60 °C for 1 h. After the addition of 10 ml of n-hexane, the top layer was transferred to a second tube and evaporated. The dried sample were resuspended in 1 ml of *n*-hexane and stored at − 20 °C until gas chromatography (GC) analysis were performed by SAVI Laboratori & Services (Italy).

Enzymatic transesterification was performed using the immobilized lipase B from *Candida antarctica* (Novozym 435, Sigma-Aldrich). The reaction mixture contained 500 µl of microbial lipids, 12 mg of Novozym 435 and 12.5 µl of methanol and was incubated at 37 °C in an orbital shaker at 160 rpm. Four addition each of 12.5 µl of methanol were done every 24 h and the reaction was stopped after 168 h by adding 2.5 ml of *n*-hexane. Samples were store at − 20 °C until Fourier Transform Infrared (FTIR) and GC analyses.

### FTIR spectroscopy and microspectroscopy

Lipid accumulation in intact cells was monitored by FTIR microspectroscopy. For this analysis, yeast cells collected at different time points were washed three times in distilled water to eliminate medium contamination. Approximately 3 μL of the cell suspensions were then deposited onto an IR transparent BaF_2_ support and dried at room temperature for at least 30 min to eliminate the excess water. FTIR absorption spectra were acquired in transmission mode, between 4.000 and 700 cm^−1^, by means of a Varian 610-IR infrared microscope coupled to the Varian 670-IR FTIR spectrometer (both from Varian Australia Pty Ltd), equipped with a mercury cadmium telluride (MCT) nitrogen-cooled detector. The variable microscope aperture was adjusted from approximately 60 μm × 60 μm to 100 μm × 100 μm. Measurements were performed at 2 cm^−1^ spectral resolution; 25 kHz scan speed, triangular apodization, and by the accumulation of 512 scan co-additions. When necessary, spectra were corrected for residual water vapour absorption.

Spectral analysis was conducted in the spectral range between 4.000 and 800 cm^−1^. To this aim, second derivative spectra were obtained following the Savitsky-Golay method (third-grade polynomial, nine smoothing points), after a binomial 13 smoothing points of the measured spectra, using the GRAMS/32 software (Galactic Industries Corporation, USA). To verify the reproducibility and reliability of the spectral results, more than three independent preparations were analysed. In the Figures, reported data are representative of the independent experiments performed.

The percentage of FAMEs produced by the lipase-catalysed reaction was determined by FTIR as described in [58]. In particular, 5 µl of the sample were deposited on the diamond element of the device for FTIR measurements in attenuated total reflection and the spectra were collected by the Varian 670-IR spectrometer (settings: 2 cm^−1^ spectral resolution, 25 kHz scan speed, 512 scan co-additions, and triangular apodization). The methyl ester content in the reaction mixture was determined from the peak intensity at ~ 1435 cm^−1^ in the second derivatives of the FTIR absorption spectra [58]. The calibration curves for the FAME quantitation were obtained using standard samples containing increasing concentrations of methyl oleate, from 0 to 100% v/v, in triolein, where a linear relationship between the methyl ester concentration and the ~ 1435 cm^−1^ peak height in the second derivative spectra was observed [58].

## Supplementary information

**Additional file 1: Table S1.** Growth rates of* L. starkeyi* in different growth conditions. Cells were grown at different % of SBP hydrolysate, with or without 6% molasses, in batch and pulsed fed-batch cultures. Growth rates has been calculated thanks to a semi-logarithmic graph (data not shown). ND: not determined; *P < 0.05; NS, no statistical significance.** Figure S1.** FTIR microspectroscopy analysis of* L. starkeyi*. The FTIR absorption spectra of* L. starkeyi* intact cells are reported at 0 and 144 h of growth in 3% SBP hydrolysate containing 6% of molasses. For comparison, spectra have been normalized to amide I band area. The assignment of the bands due to the biomolecules discussed in the text is reported.** Figure S2.** Linear relation between OD660 and lipid accumulation measured as CH stretching band area (A) and ester C=O (B) by Fourier transform infrared (FTIR) of* L. starkeyi* cells growing in 3% SBP hydrolysate blended with 1%, 2% or 6% of molasses in batch and pulsed-fed batch cultures. Tables show the values corresponding to the slope of the regression lines and to the coefficients of determination (R2).** Figure S3.** Sucrose, glucose, arabinose, acetic acid and lactic acid concentrations during growth of* L. starkeyi* in SBP hydrolysate generated at 3% TS fed with pulses of molasses.** Figure S4.** Lipase catalysed transesterification of lipids produced by* L. starkeyi* cultivated in SBP fed with molasses (pattern E). 500 µl of lipids were incubated with 12 mg of Novozym 435 at 37°C and 160 rpm. 2.5% of methanol were added at time 0, 24, 48 and 72 h (indicated by arrows).

## Data Availability

All data generated or analysed during this study are included in this published article and its supplementary information file.

## Abbreviations

C/N: Carbon/nitrogen
GHG: Greenhouse gas
TAGs: Triacylglycerols
FAAEs: Fatty acid alkyl esters
SCOs: Single-cell oils
SBP: Sugar beet pulp
SBM: Sugar beet molasses
*T. fermentas*: *Trichosporon fermentas*
*L. starkeyi*: *Lipomyces starkeyi*
TS: Total solid
OD_660_: Optical density at 660 nm
AI: Amide I
FTIR: Fourier transform infrared
CDW: Cellular dry weight
FAMEs: Fatty acid methyl esters
NOx: Nitrogen oxides
M: Monounsaturated fatty acids
P: Polyunsaturated fatty acids
S: Saturated fatty acids
rpm: Rotations per minute
YPD: Yeast extract peptone d-glucose
HPLC: High performance liquid chromatography
GC: Gas chromatography
MCT: Mercury cadmium telluride
