# Supplementary material for: Conversion of sugar beet residues into lipids by Lipomyces starkeyi for biodiesel production
Source: Microb Cell Fact. 2020 Nov 10;19:204. doi: 10.1186/s12934-020-01467-1 (PMC7653891; doi:10.1186/s12934-020-01467-1)
Supplement: Supplementary file 1 — Additional file 1: Table S1. Growth rates of L. starkeyi in different growth conditions. Cells were grown at different % of SBP hydrolysate, with or without 6% molasses, in batch and pulsed fed-batch cultures. Growth rates has been calculated thanks to a semi-logarithmic graph (data not shown). ND: not determined; *P < 0.05; NS, no statistical significance. Figure S1. FTIR microspectroscopy analysis of L. starkeyi. The FTIR absorption spectra of L. starkeyi intact cells are reported at 0 and 144 h of growth in 3% SBP hydrolysate containing 6% of molasses. For comparison, spectra have been normalized to amide I band area. The assignment of the bands due to the biomolecules discussed in the text is reported. Figure S2. Linear relation between OD660 and lipid accumulation measured as CH stretching band area (A) and ester C=O (B) by Fourier transform infrared (FTIR) of L. starkeyi cells growing in 3% SBP hydrolysate blended with 1%, 2% or 6% of molasses in batch and pulsed-fed batch cultures. Tables show the values corresponding to the slope of the regression lines and to the coefficients of determination (R2). Figure S3. Sucrose, glucose, arabinose, acetic acid and lactic acid concentrations during growth of L. starkeyi in SBP hydrolysate generated at 3% TS fed with pulses of molasses. Figure S4. Lipase catalysed transesterification of lipids produced by L. starkeyi cultivated in SBP fed with molasses (pattern E). 500 µl of lipids were incubated with 12 mg of Novozym 435 at 37°C and 160 rpm. 2.5% of methanol were added at time 0, 24, 48 and 72 h (indicated by arrows). [file 12934_2020_1467_MOESM1_ESM.docx]

**Additional information**

**Conversion of sugar beet residues into lipids by *Lipomyces starkeyi* for biodiesel production**

Francesca Martani^1#^, Letizia Maestroni^1#^, Mattia Torchio^1^, Diletta Ami^1^, Antonino Natalello^1^, Marina Lotti^1^, Danilo Porro^1^, Paola Branduardi^1*^

^1^Department of Biotechnology and Biosciences, University of Milano Bicocca, 20126 Milan, Italy

* Corresponding author: [paola.branduardi@unimib.it](mailto:paola.branduardi@unimib.it)

# These authors contributed equally to this work.


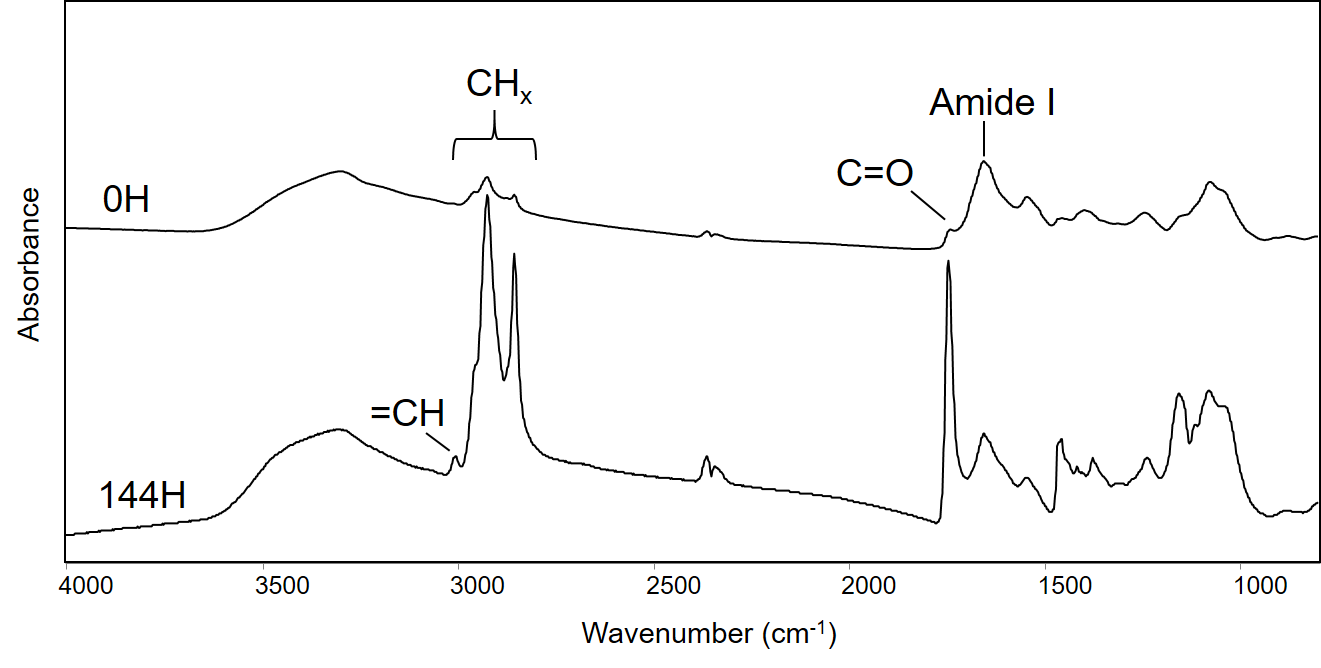


**Figure S1.** FTIR microspectroscopy analysis of *L. starkeyi*. The FTIR absorption spectra of *L. starkeyi* intact cells are reported at 0 and 144 h of growth in 3% SBP hydrolysate containing 6% of molasses. For comparison, spectra have been normalized to amide I band area. The assignment of the bands due to the biomolecules discussed in the text is reported.

**
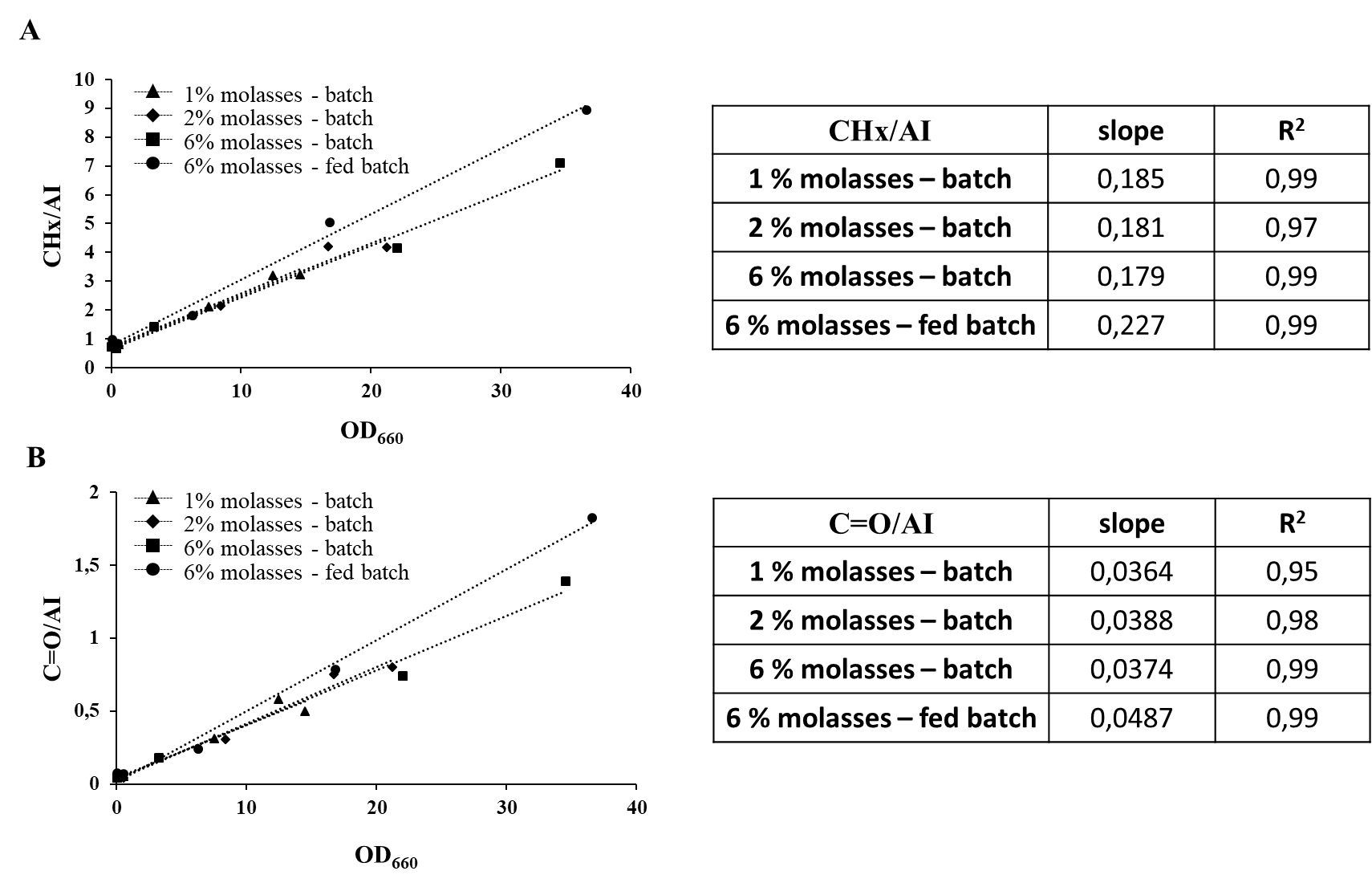
**

**Figure S2**. Linear relation between OD_660_ and lipid accumulation measured as CH stretching band area (**A**) and ester C = O (**B**) by Fourier transform infrared (FTIR) of *L. starkeyi* cells growing in 3 % SBP hydrolysate blended with 1 %, 2 % or 6 % of molasses in batch and pulsed-fed batch cultures. Tables show the values corresponding to the slope of the regression lines and to the coefficients of determination (R^2^).

**Figure S3.** Sucrose, glucose, arabinose, acetic acid and lactic acid concentrations during growth of *L. starkeyi* in SBP hydrolysate generated at 3 % TS fed with pulses of molasses.


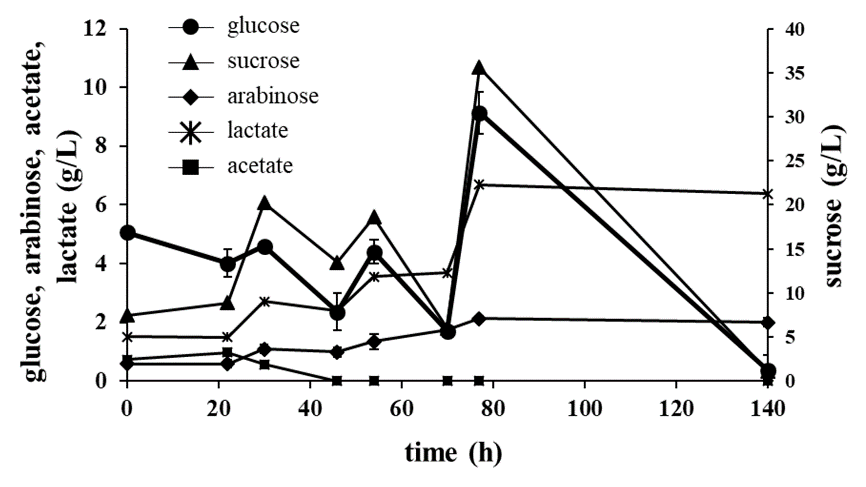


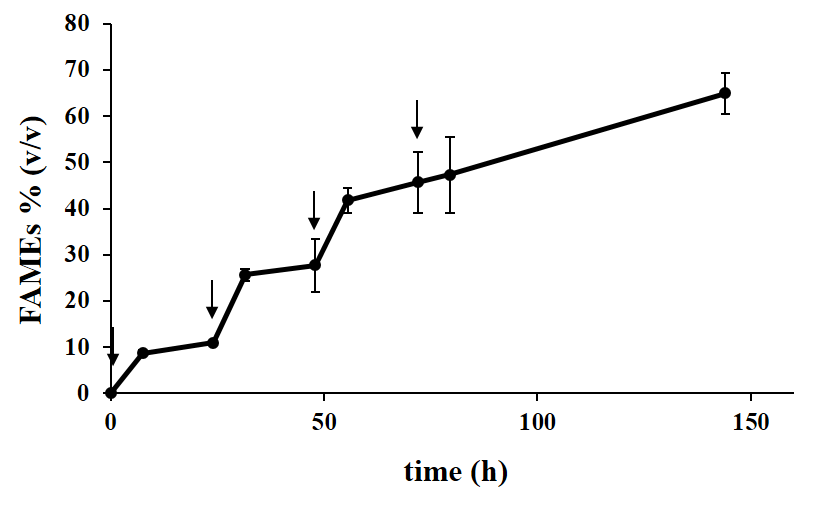


**Figure S4.** Lipase catalyzed transesterification of lipids produced by *L. starkeyi* cultivated in SBP fed with molasses (pattern E). 500 µl of lipids were incubated with 12 mg of Novozym 435 at 37°C and 160 rpm. 2.5 % of methanol were added at time 0, 24, 48 and 72 h (indicated by arrows).

| Cultivation mode | Molasses | SBP | µ | *P* |
| --- | --- | --- | --- | --- |
| Batch | - | 1 % | ND |  |
|  | - | 2 % | 0.071 ± 0.004 | a |
|  | - | 3 % | 0.079 ± 0.003 | b |
|  | - | 4 % | 0.081 ± 0.003 | b |
|  | - | 5 % | 0.078 ± 0.003 | b |
|  | - | 6 % | ND |  |
|  | - | 7 % | ND |  |
|  | 6 % | 3 % | 0.089 ± 0.002 | a’ |
| Pulsed-fed batch | 6 % | 3 % | 0.096 ± 0.003 | b’ |

**Table S1.** Growth rates of *L. starkeyi* in different growth conditions. Cells were grown at different % of SBP hydrolysate, with or without 6 % molasses, in batch and pulsed fed-batch cultures. Growth rates has been calculated thanks to a semi-logarithmic graph (data not shown). ND: not determined; statistical significance is expressed by letters. *P* < 0.05.
